# Increased expression of miR-221 is associated with shorter overall survival in T-cell acute lymphoid leukemia

**DOI:** 10.1186/2162-3619-2-10

**Published:** 2013-04-08

**Authors:** Hamilton L Gimenes-Teixeira, Antonio R Lucena-Araujo, Guilherme A dos Santos, Dalila L Zanette, Priscila S Scheucher, Luciana C Oliveira, Leandro F Dalmazzo, Wilson A Silva-Júnior, Roberto P Falcão, Eduardo M Rego

**Affiliations:** 1 Department of Internal Medicine, Division of Hematology/Oncology, University of São Paulo, Ribeirão Preto, Brazil; 2 Department of Genetics, Medical School of Ribeirão Preto and National Institute of Cell Based Therapy, University of São Paulo, Ribeirão Preto, Brazil; 3 Medical School of Ribeirao Preto, University of São Paulo, Av. Bandeirantes, 3900, Ribeirao Preto, SP, 14048-900, Brazil

**Keywords:** T-cell acute lymphoid leukemia, miRNA, CD56, Treatment outcome

## Abstract

**Background:**

CD56 expression has been associated with a poor prognosis in lymphoid neoplasms, including T-cell acute lymphoblastic leukemia (T-ALL). MicroRNAs (miRNAs) play an important role in lymphoid differentiation, and aberrant miRNA expression has been associated with treatment outcome in lymphoid malignancies. Here, we evaluated miRNA expression profiles in normal thymocytes, mature T-cells, and T-ALL samples with and without CD56 expression and correlated microRNA expression with treatment outcome.

**Methods:**

The gene expression profile of 164 miRNAs were compared for T-ALL/CD56^+^ (n=12) and T-ALL/CD56^-^ (n=36) patients by Real-Time Quantitative PCR. Based on this analysis, we decided to evaluate miR-221 and miR-374 expression in individual leukemic and normal samples.

**Results:**

miR-221 and miR-374 were expressed at significantly higher levels in T-ALL/CD56^+^ than in T-ALL/CD56^-^ cells and in leukemic blasts compared with normal thymocytes and peripheral blood (PB) T-cells. Age at diagnosis (15 or less *vs* grater than 15 years; HR: 2.19, 95% CI: 0.98-4.85; *P*=0.05), miR-221 expression level (median value as cut off in leukemic samples; HR: 3.17, 95% CI: 1.45-6.92; *P*=0.004), and the expression of CD56 (CD56^-^*vs* CD56^+^; HR: 2.99, 95% CI: 1.37-6.51; *P*=0.006) were predictive factors for shorter overall survival; whereas, only CD56 expression (HR: 2.73, 95% CI: 1.03-7.18; *P*=0.041) was associated with a shorter disease-free survival rate.

**Conclusions:**

miR-221 is highly expressed in T-ALL and its expression level may be associated with a poorer prognosis.

## Background

Neural-cell adhesion molecule (N-CAM; CD56) is a known marker of natural killer (NK) cells [1]. The two best characterized forms of NK-cell malignancy are the aggressive NK-cell leukemia (ANKL) and the extra nodal NK-cell lymphoma, nasal type (ENKL) [2,3]. However, CD56 is also expressed on a subset of normal T cells and occasionally on blasts in T-cell acute lymphoblastic leukemia (T-ALL) [4]. The expression status of CD56 identified a subgroup of patients with T-ALL who did not respond well to therapy [4,5]. From an ontogenetic point of view, NK cells arise from T/NK bi-potential common progenitors [6,7]; therefore, NK-cells are functionally and phenotypically very similar to T-cells, particularly cytotoxic T-cells.

MicroRNAs (miRNAs) play an important role in lymphoid differentiation and in innate immune response [8-10], and aberrant miRNA expression has been associated with treatment outcome in hematological malignancies [11-13]. Wang *et al.* demonstrated that miR-378 and miR-30e are suppressors of NK cell cytotoxicity and their expression is regulated by IFNα [14]. Furthermore, Chiaretti *et al*. recently described a large set of myeloid-related miRNAs overexpressed in a subset of adult T-ALL patients; interestingly, the “myeloid-like” cases presented with higher expression levels of miR-223 and poorer prognoses compared with T-ALL patients without overexpression of myeloid-related genes [15].

Some miRNAs may be relevant in T-ALL leukemogenesis. Mavrakis *et al.* identified five miRNAs (miR-19b, miR-20a, miR-26a, miR-92, and miR-223) in a human T-ALL library that were capable of promoting T-ALL development in a mouse model [16]. Moreover, these miRNAs acted synergistically with tumor suppressor genes implicated in the pathogenesis of T-ALL [16]. In the present study, we compared expression of 164 miRNAs in T-ALL blasts with and without CD56 expression and correlated these profiles with T-cell development and treatment outcome.

## Results and discussion

**Figure 1:**
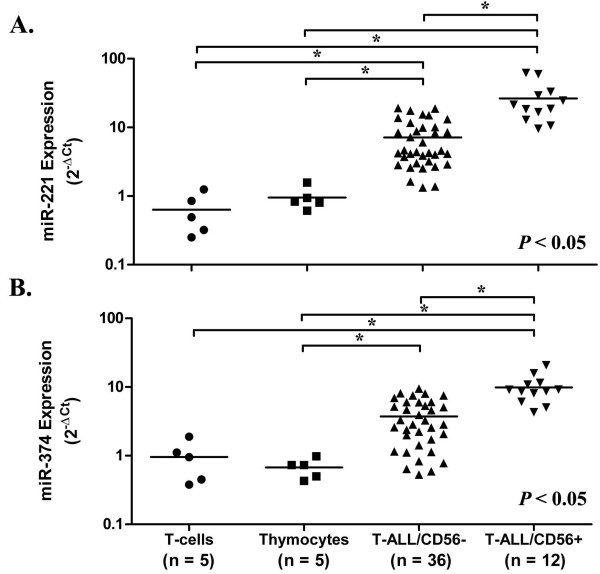
**Quantitative analysis of miR-221 (A) and miR-374 (B) expression in samples from T-ALL patients, T-cells from normal peripheral blood, and CD3**^**+**^**/CD4**^**+**^**/CD8**^**+**^**thymocytes.** Leukemic samples from 48 T-ALL patients were obtained from cell banks. The expression of miRNAs was quantified by Real-Time Quantitative PCR (RQ-PCR). The horizontal bars represent the mean of miRNA expression calculated by 2^-ΔCt^. T-ALL samples were categorized according to presence of the CD56 cell marker: 12 samples were positive (T-ALL/CD56^+^) and 36 were negative (T-ALL/CD56^-^). Asterisks indicate significant differences between groups (ANOVA test followed by a Dunn’s post-test).

Initially, we identified 56 miRNAs with higher expression and 35 with lower expression levels in the T-ALL/CD56^+^ group compared with the T-ALL/CD56^-^ group (Additional file 1: Table S1). Expression of miR-221 and miR-374 were higher in T-ALL/CD56^+^ compared with T-ALL/CD56^-^ cells, with 271- and 181-fold changes, respectively, and were chosen for further analyses. Irrespective of CD56 expression, miR-221 (Figure 1A) and miR-374 (Figure 1B) expression were higher in leukemic blasts compared with normal peripheral blood (PB) T-cells and thymocytes. By adopting the upper limits of the 95% confidence intervals of miR-221 and miR-374 expression in the normal T/CD3^+^ samples as cut-offs, 48/48 (100%) and 38/47 (81%) of the T-ALL samples, respectively, had expression higher than the cut-off values.

We did not detect differences in miR-221 and miR-374 expression when comparing triple positive thymocytes (CD3^+^/CD4^+^/CD8^+^) and mature T-cells in PB of healthy subjects. Our results do not corroborate the study by Kirigin *et al*. [8], which analyzed miRNAs in different stages of T-cell differentiation in the murine thymus and bone marrow using next generation sequencing. The authors demonstrated that there was greater expression of miR-221 and miR-222 in early stages compared with mature thymocytes. However, the comparison between the two studies must be regarded with caution because Kirigin *et al*. [8] have used a more complex strategy of cell sorting, and analyzed T-cell subsets that do not completely overlap with those of our study. The double positive (DP) thymocytes in their study had the immunophenotype CD90^+^CD4^+^CD8^+^CD3^low^ and were compared with CD4 single positive (SP) (CD90^+^CD4^+^CD8^-^CD24^low^CD3^high^), and CD8SP (CD90^+^CD42CD8^+^CD24^low^CD3^high^) subsets. In addition, the most significant difference in miR-221 expression demonstrated by Kirigin *et al.*[8] was between double negative four (DN4, CD90^+^CD4^-^CD8^-^CD3^low^CD44^-^CD25^-^) thymocytes and CD4SP and CD8SP mature T-cells [8], and such analysis was not performed in the present study.

miR-221 is up-regulated in several human malignancies; whereas, there are few reports associating miR-374 with tumorigenesis. Nevertheless, the mechanisms leading to the higher expression of these miRNAs are unknown. Recently, Santhekadur *et al*. [17] demonstrated that RNA Induced Silencing Complex (RISC) proteins SND1 and AEG-1 induce miR-221 expression in a NFκB dependent way in liver cancer cells. An alternative explanation would be that extracellular stimuli may interfere with miRNAs expression. With this regard, Davis *et al*. [18] demonstrated that miR-221 is a mediator of Platelet Derived Growth Factor (PDGF) signaling through modulation of p27^Kip1^. Remarkably, high levels of PGDF signaling were described as a decisive factor for proliferation and survival in cytotoxic T and natural killer cell neoplasms [19].

The transcription factor BCL11B has been identified as a putative target of miR-221 and it has been previously demonstrated that BCL11B expression is required to repress natural killer cell–associated genes and essential for T lineage commitment [20-22]. Overexpression of BCL11B was reported in T-ALL [23] and in adult T-cell leukemia/lymphoma [24] and its silencing in Molt-4 cells was associated with increased expression of the antiapoptotic protein Bcl-2 [20]. Previously reported miR-221 targets include the cell cycle regulators CDKN1B/p27^Kip1^ and CDKN1C/p57^Kip2^[25-27]. Le Sage *et al*. [25] demonstrated that cancer cell lines require high expression of miR-221/222 to maintain low p27^kip1^ levels and continuous proliferation. Therefore, it is conceivable that miR-221 acts as an oncogene through inhibition of p27^kip1^ in T-ALL. Another known target of miR-221 is the receptor c-KIT. Fellicetti *et al*. [28] showed an inverse correlation between miR-221 and miR-222 expression and c-KIT protein levels during melanoma progression. In addition, c-KIT is rapidly up-regulated following NOTCH signaling in T-cell development, and the development of primitive cells into non T-cell fates (NK or myeloid) was found to be c-kit independent [28].

**Figure 2:**
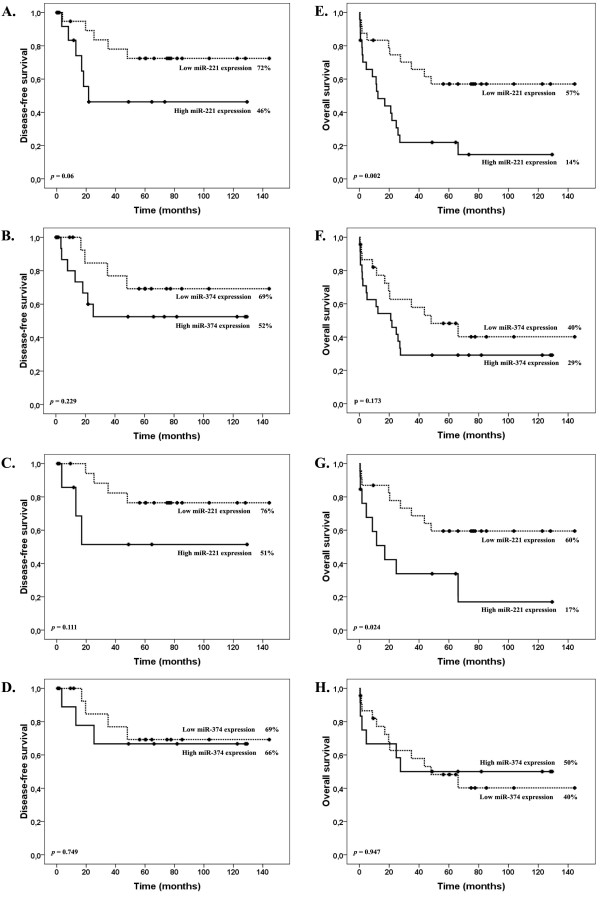
**Disease-free survival (2A-2D) and overall survival (2E-2H) in T-ALL patients according to miR-221 and miR-374 expression.** 2**A**-2**B** and 2**E**-2**F** represent analyses in the whole population; whereas, 2**C**-2**D** and 2**G**-2**H** represent analyses in only T-ALL/CD56^-^ patients.

For the analysis of the relevance of miR-221 and miR-374 expression levels in the response to treatment, we arbitrarily divided T-ALL patients in low (≤ median value in all T-ALL samples) or high (> than the median value) expression groups. The main clinical and laboratory features at diagnosis are summarized in Table 1. The only significant differences between patients with high *versus* low expression of miR-221 and miR-374 were higher platelet counts in patients expressing higher levels of miR-221 (*P* = 0.009) and miR-374 (*P* = 0.03) and a higher frequency of CD34^+^ cases in the group with higher miR-221 expression (*P* = 0.042). The mean follow-up among survivors was 27 months (range, 12 to 42 months). The complete hematological remission (CR) rate for the whole population was 66% and did not differ between high- and low- miR-221 (54% *versus* 79%; *P* = 0.125) and miR-374 (62% *versus* 69%; *P* = 0.76) expression groups. In the whole population, high miR-221 patients had a lower disease-free survival (DFS) (46%) compared with low miR-221 patients (72%, *P* = 0.06; Figure 2A); while, miR-374 did not impact the DFS (69% *versus* 52%, *P* = 0.229; Figure 2B). When the analysis was restricted to T-ALL/CD56^-^ patients, the DFS did not differ between low and high miR-221 (76% *versus* 51%, *P* = 0.111; Figure 2C) and miR-374 groups (69% *versus* 66%, *P* = 0.749; Figure 2D).

**Table 1:** Clinical and immunophenotypic features of T-ALL patients at diagnosis according to expression of miR-221 and miR-374

**Patient characteristics**	**miR-221**			**miR-374**		
	**High expression**	**Low expression**	***P*-value**	**High expression**	**Low expression**	***P-*value**
Age^1^, years	22.8 (1 – 66)	17.4 (2 – 44)	0.22	23.6 (1 – 66)	16.8 (2 – 44)	0.49
Gender (M/F)	24(18/6)	24 (21/3)	0.46	24(21/3)	23(17/6)	0.12
Mediastinal mass (%)	14/24(58)	6/24 (25)	0.15	15/24 (62.5)	5/23(22)	0.13
Lymphadenopathy (%)	15/24(62.5)	21/24 (87.5)	0.26	16/24 (67)	19/23(83)	0.24
Splenomegaly (%)	16/24(67)	16/24 (67)	0.66	17/24 (71)	14/23(61)	0.6
Hepatomegaly (%)	15/24(62.5)	16/24 (67)	0.99	17/24 (71)	13/23(56.5)	0.44
CNS involvement (%)	1/24(4)	2/24 (8)	0.56	2/24 (8)	1/23(4)	0.53
Blasts BM^1^ (%)	77.3 (20 – 100)	90.5 (28 – 100)	0.09	77.6 (20 – 100)	90.7 (28 – 100)	0.64
Blasts PB^1^ (%)	63.4 (0 – 100)	78.5 (10 – 100)	0.18	61.5 (0 – 100)	86 (10 – 100)	0.45
Hemoglobin^1^ (g/l)	10.4 (3.6 – 15.6)	10.4 (4.6 – 17.1)	0.97	10.1 (3.6 – 15.8)	10.8 (5.3 – 17.1)	0.4
WBC^1^ count × 10^9^/l	104 (4.6 – 314)	202.3 (4.5 – 790)	0.06	118 (4.5 – 781)	196 (18 – 789)	0.61
Platelets^1^ × 10^9^/l	149.4 (9 – 652)	48.8 (9 – 185)	0.009*	140 (9 – 652)	55.8 (9 – 231)	0.03*
Pre thymic (%)	7/24(29)	4/24(17)	0.31	6/24 (25)	5/23(22)	0.81
Thymic (%)	7/24(29)	10/24(42)	0.54	9/24 (37.5)	7/23(30)	0.51
Mature T (%)	8/24(33)	8/24(33)	0.98	7/24 (29)	9/23(39)	0.37
CD34 (%)	16/24(67)	8/24(33)	0.042*	13/24 (54)	11/23(48)	0.56
CD56 (%)	11/24(46)	1/24(4)	0.001*	12/24 (50)	1/23(4)	<0.001*
TdT (%)	18/24(75)	21/24(87)	0.46	17/24 (71)	21/23(91)	0.28

CNS: central nervous system; WBC: white blood cell; PB: peripheral blood; BM: bone marrow; cCD3: cytoplasmic CD3; sCD3: surface CD3; TdT: terminal deoxynucleotidyl transferase. *Significant *P*-value.

^1^ Values represent mean (range).

Patients with high miR-221 expression had significantly lower 5-year OS rates compared with those with low miR-221 expression (14% *versus* 57%, *P* = 0.002; Figure 2E). Among T-ALL/CD56^-^ patients, those with higher miR-221 expression had lower OS (17% *versus* 60%, *P* = 0.024; Figure 2G). No difference in OS was observed based on miR-374 expression when considering the whole population (40% *versus* 29% in low- *versus* high-miR374 groups, *P* = 0.173; Figure 2F) or just T-ALL/CD56^-^ patients (40% *versus* 50% in low- *versus* high-miR374 groups, *P* = 0.947; Figure 2H). In the univariate analysis, expression of miR-221 (HR: 3.17, 95% CI: 1.45 to 6.92; *P* = 0.004), CD56 expression (HR: 2.99, 95% CI: 1.37 to 6.51; *P* = 0.006), and age (HR: 2.19, 95% CI: 0.98 to 4.85; *P* = 0.05) were associated with poorer OS; whereas, only CD56 expression was predictive of poorer DFS (HR: 2.73, 95% CI: 1.03 to 7.18; *P* = 0.041). Nonetheless, these factors were not independent after adjustment (Table 2).

**Table 2:** Univariate and multivariate analyses for CR, OS, and DFS in 48 patients with T-ALL

**End point**	**Model variables**	**Univariate analysis**					**Multivariate analysis**				
		**HR**	**OR**	**95% CI**		***P*-value**	**HR**	**OR**	**95% CI**		***P*-value**
CR	miR-221 expression (low *vs* high)		0.63	0.17	2.39	0.507		1.71	0.26	10.9	0.571
	WBC counts (≤ 30×10^9^/L *vs* > 30×10^9^/L)		1.55	0.32	7.41	0.581		0.92	0.11	7.15	0.94
	Age at diagnosis (≤ 15 *vs* > 15)		1.68	0.43	6.54	0.454		2.63	0.53	12.9	0.234
	CD56 expression (CD56^-^*vs* CD56^+^)		0.33	0.08	1.38	0.133		0.14	0.01	1.2	0.073
OS	miR-221 expression (low *vs* high)	3.17		1.45	6.92	0.004	2.31		0.92	5.81	0.074
	WBC counts (≤ 30x10^9^/L *vs* > 30×10^9^/L)	0.69		0.29	1.64	0.412	1.41		0.53	3.7	0.481
	Age at Diagnosis (≤ 15 *vs* > 15)	2.19		0.98	4.85	0.05	2.2		0.96	5	0.059
	CD56 expression (CD56^-^*vs* CD56^+^)	2.99		1.37	6.51	0.006	2.04		0.76	5.46	0.155
DFS	miR-221 expression (low *vs* high)	1.87		0.89	3.92	0.093	1.54		0.57	4.17	0.391
	WBC counts (≤ 30×10^9^/L *vs* > 30×10^9^/L)	1.76		0.81	3.82	0.148	1.81		0.65	5.01	0.25
	Age at diagnosis (≤ 15 *vs* > 15)	0.77		0.37	1.6	0.488	0.89		0.42	1.9	0.778
	CD56 expression (CD56^-^*vs* CD56^+^)	2.73		1.03	7.18	0.041	2.25		0.69	7.28	0.173

Abbreviations: *OS*: overall survival; *DFS*: disease-free survival; *CR*: complete remission; *HR*, Hazard ratio; *OR*: odds ratio; *WBC*: white blood count.

We selected the T-ALL/CD56^+^ and CD56^-^ subgroups based on previous studies by Dalmazzo *et al*. [4] and Fischer *et al*. [29], which suggest that CD56 identifies a subgroup of patients with distinct immunophenotypic and clinical features, such as higher resistance to therapy and older age. Nevertheless, after risk adapted treatment, the prognostic impact of CD56 expression was not significant in the study by Fisher *et al*. [29] in contrast to the results reported by Dalmazzo *et al*. [4]. We must point out that 47/48 (98%) patients reported here were also included in the study of Dalmazzo *et al*. [4], although the follow-up data were updated.

Our results suggest that miR-221 may be a useful biomarker in T-ALL. Patients with T-ALL/CD56^-^with high expression of miR-221, as well as those with T-ALL/CD56^+^ presented shorter OS and, therefore may require a more careful monitoring of the response to treatment and/or may be considered candidates to more intensive therapy. However, our conclusions are limited by the sample size and need to be further investigated in a larger cohort.

## Methods

### Patients

Forty-eight bone marrow samples from T-ALL patients were analyzed. All cases were diagnosed in the Hematology Laboratory of the University Hospital of the Medical School of Ribeirão Preto, University of São Paulo from May 1997 to April 2008. The diagnosis of T-ALL was established based on the World Health Organization criteria [30]. Patients were treated according to the HyperCVAD [31] (n = 6), Berlin-Frankfurt-Munich 90 (BFM-90) [32] (n = 16), or Brazilian Childhood Leukemia Treatment Group – Acute Lymphoid Leukemia 99 (GBTLI-ALL99) [33] (n = 20) protocols. Six patients were excluded from survival analyses; one patient died before the beginning of treatment and the others five had no clinical data available. This study was approved by the local Ethics Committee (process #7147/2005).

### Purification of thymocytes and peripheral blood T-cells

Thymic samples were obtained as surgical tissue discards from five pediatric patients (aged 2 days to 5 years) undergoing cardiac surgery at the University Hospital of the Medical School of Ribeirão Preto. Thymocytes were isolated by cutting the thymic lobes into small pieces and forcing them through plastic mesh. Triple positive thymocytes (CD3^+^/CD4^+^/CD8^+^) were purified by immunomagnetic separation (Miltenyi Biotec) after labeling with anti-CD4 and anti-CD8 antibodies, and then passed through a lymphocyte separation column (Miltenyi Biotec, Germany) for the positive selection of labeled cells. After isolation, all samples were more than 95% pure.

Peripheral blood mononuclear cells (PBMCs) from five healthy donors were harvested by centrifugation on Ficoll-Hypaque (Sigma Aldrich, USA) density gradients, and PB T/CD3^+^ cells were isolated using magnetic-activated cell sorting (MACS; Miltenyi Biotec, Germany), according to the manufacturer’s protocol. The homogeneity of PB T/CD3^+^ cells was confirmed by flow cytometry, and the purity was greater than 85% in all samples.

### Immunophenotyping

Immunophenotyping of the blasts was performed by flow cytometry. The technique and the panel of fluorochrome-conjugated monoclonal antibodies were described previously [4]. We also expanded the panel and analyzed the expression of TdT, CD34, cytoplasmic CD3 (cCD3), surface CD3 (sCD3), CD4, and CD8 in leukemic cells. The minimum threshold for a positive reaction to a given antibody was defined as 20% of blasts positive for the respective antigen.

### RNA extraction and real-time quantitative PCR of miRNAs

Total RNA from leukemic samples and healthy donors was isolated using Trizol reagent (Invitrogen, Carlsbad, CA, USA). Complementary DNA (cDNA) was synthesized from 1 μg of total RNA using a High Capacity cDNA reverse transcription Kit (Applied BioSystems, Foster City, CA, USA), following the manufacturer’s instructions. The TaqMan® MicroRNA Assays Human Panel (Applied BioSystems, Foster City, CA, USA) was employed to assess the expression levels of 164 miRNAs in leukemic samples. Due to the limited amount of RNA, we pooled the samples according to the immunophenotype (T-ALL/CD56^+^*versus* T-ALL/CD56^-^) and performed a preliminary analysis comparing the two groups. Comparisons with coefficients of variation greater than 5% were excluded. We defined a strength cut-off for differential expression as ≥ 4 and ≤ 0.25. The fold change was calculated using the comparative cycle threshold (Ct) method, in which the geometric mean of expression of RNUs 6B, 19, 38B and 66 was used for normalization.

Based on this preliminary analysis, we decided to evaluate expression of miR-221 and miR-374 in individual T-ALL, normal thymic, and PB samples. *TaqMan*-based Real Time Quantitative Polymerase Chain Reaction (RQ-PCR) assays were performed using specific RT-stem-looped primers and probes (Applied BioSystems, Foster City, CA, USA). All reactions were carried out in duplicate.

### Statistical analysis

The median values of miR-221 and miR-374 expression in leukemic samples were used as references to classify T-ALL patients into high and low expression groups. Student’s t and Fisher’s exact tests were employed to compare differences between the groups. Overall survival (OS) was estimated by the Kaplan–Meier method. Differences among groups were compared by log-rank test. Multivariate regression analysis was performed for OS using the Cox proportional hazards model. All *P*-values were two sided. The level of significance was set at 5%. All the statistical tests were performed using the Statistical Package for the Social Sciences (SPSS) v.17.0 software (SPSS Inc, Chicago, IL, USA) and Stata Statistic/Data Analysis 9.1 (Stata Corporation, College Station, TX, USA).

## Competing interest

The authors declare that they have no competing interest.

## Authors’ contributions

HLGT, ARLA and GAS performed experiments, analyzed and interpreted data and drafted the article. DLZ and PSS performed experiments and collected data. ARLA and LCO performed the statistical analyses. LFD provided the samples and clinical data. ARLA and EMR designed the experiments, interpreted data and reviewed the manuscript. All authors read and approved the final manuscript.

## Supplementary Material

Additional file 1: Table S1 Relative expression of miRNAs in T-ALL/CD56^+^ pooled samples. Click here for file
