# Supplementary material for: Increased expression of miR-221 is associated with shorter overall survival in T-cell acute lymphoid leukemia
Source: Exp Hematol Oncol. 2013 Apr 8;2:10. doi: 10.1186/2162-3619-2-10 (PMC3637292; doi:10.1186/2162-3619-2-10)
Supplement: Additional file 1: Table S1 — Relative expression of miRNAs in T-ALL/CD56+ pooled samples. [file 2162-3619-2-10-S1.doc]

**Supplementary Table 1.** Relative expression of miRNAs in T-ALL/CD56^+^ pooled samples.

| ***miRNA*** | ***Relative Expression^1^*** | ***Genomic Localization*** | ***Gene Locus*** | ***DNA***  ***Strand*** | ***Organization*** |
| --- | --- | --- | --- | --- | --- |
| ***miR-221*** | **271,40** | **Intergenic** | **Xq11.3** | **-** | **Cluster** |
| ***miR-374*** | **105,84** | **Intergenic** | **Xq13.2** | **-** | **Cluster** |
| ***miR-135a*** | **15,74** | **Intergenic** | **3p21.1** | **-** | **Single** |
| ***miR-199a*** | **7,90** | **Intron** | **19p13.2** | **-** | **Single** |
| ***miR-133b*** | **5,71** | **Intergenic** | **6p12.2** | **+** | **Cluster** |
| ***miR-126*** | **5,60** | **Intron** | **9q34.3** | **+** | **Single** |
| ***miR-145*** | **5,30** | **Intergenic** | **5q33.1** | **+** | **Cluster** |
| ***miR-199b*** | **5,24** | **Intron** | **9q34.11** | **-** | **Single** |
| ***miR-223*** | **5,13** | **Intergenic** | **Xq12** | **+** | **Single** |
| ***miR-146*** | **4,78** | **Intergenic** | **5q33.3** | **+** | **Single** |
| ***miR-125a*** | **4,40** | **Intergenic** | **19q13.33** | **+** | **Cluster** |
| *miR-182* | 3,77 | Intergenic | 7q32.2 | - | Cluster |
| *miR-34a* | 3,74 | Intergenic | 1p36.23 | - | Single |
| *miR-99a* | 3,52 | Intron | 21q21.1 | + | Cluster |
| *miR-330* | 2,98 | Intron | 19q13.32 | - | Single |
| *Let-7e* | 2,73 | Intergenic | 19q13.33 | + | Cluster |
| *miR-338* | 2,68 | Intron | 17q25.3 | - | Cluster |
| *miR-222* | 2,40 | Intergenic | Xp11.3 | - | Cluster |
| *miR-211* | 2,31 | Intron | 15q13.3 | - | Single |
| *miR-132* | 2,12 | Intergenic | 17p13.3 | - | Cluster |
| *miR-103* | 1,82 | Intron | 5q35.1 | - | Single |
| *miR-30a-3p* | 1,80 | Intergenic | 6q13 | - | Single |
| *miR-21* | 1,71 | Intergenic | 17q23.1 | + | Single |
| *miR-98* | 1,68 | Intron | Xp11.22 | - | Cluster |
| *miR-339* | 1,66 | Intron | 7p22.3 | - | Single |
| *miR-130a* | 1,66 | Intergenic | 11q12.1 | + | Single |
| *miR-23a* | 1,66 | Intergenic | 19p13.12 | - | Cluster |
| *miR-133a* | 1,65 | Intron | 18q11.2 | - | Cluster |
| *miR-214* | 1,62 | Intron | 1q24.3 | - | Cluster |
| *miR-140* | 1,59 | Intron | 16q22.1 | + | Single |
| *miR-128a* | 1,58 | Intron | 2q21.3 | + | Single |
| *miR-27b* | 1,49 | Intron | 9q22.32 | + | Cluster |
| *miR-182* | 1,40 | Intergenic | 7q32.2 | - | Cluster |
| *miR-198* | 1,37 | Intron | 3q13.33 | - | Single |
| *miR-29a* | 1,33 | Intergenic | 7q32.3 | - | Cluster |
| *miR-128b* | 1,33 | Intron | 3p22.3 | + | Single |
| *miR-26b* | 1,33 | Intron | 2q35 | + | Single |
| *miR-181a* | 1,32 | Intergenic | 1q31.3 | - | Single |
| *miR-28* | 1,28 | Intron | 3q28 | + | Single |
| *miR-30d* | 1,26 | Intergenic | 8q24.22 | - | Cluster |
| *miR-213* | 1,25 | Intergenic | 1q31.3 | - | Single |
| *miR-342* | 1,23 | Intron | 14q32.2 | + | Single |
| *Let-7g* | 1,22 | Intron | 3p21.1 | - | Single |
| *miR-20* | 1,16 | Intron | 13q31.3 | + | Cluster |
| *miR-296* | 1,15 | Intergenic | 20q13.32 | - | Cluster |
| *miR-181c* | 1,15 | Intergenic | 19p13.12 | + | Cluster |
| *miR-197* | 1,15 | Intergenic | 1p13.3 | + | Single |
| *miR-30b* | 1,13 | Intergenic | 8q24.22 | - | Cluster |
| *miR-106a* | 1,12 | Intron | Xq26.2 | - | Cluster |
| *miR-181b* | 1,09 | Intergenic | 1q31.3 | - | Cluster |
| *miR-186* | 1,08 | Intron | 1p31.1 | - | Single |
| *miR-210* | 1,04 | Intergenic | 11p15.5 | - | Single |
| *Let-7d* | 1,03 | Intergenic | 9q22.32 | + | Cluster |
| *miR-92* | 1,01 | Intron | 13q31.3 | + | Cluster |
| *miR-328* | 1,01 | Intron | 16q22.1 | - | Single |
| *miR-195* | 1,01 | Intergenic | 17p13.1 | - | Cluster |
| *miR-155* | 1,00 | Intergenic | 21q21.3 | + | Single |
| *miR-331* | -1,02 | Intergenic | 12q22 | + | Single |
| *miR-29b* | -1,03 | Intergenic | 7q32.3 | - | Cluster |
| *miR-320* | -1,04 | Intergenic | 8p21.3 | - | Single |
| *Let-7a* | -1,05 | Intergenic | 9q22.32 | + | Cluster |
| *miR-184* | -1,08 | Intergenic | 15q25.1 | + | Single |
| *miR-16* | -1,11 | Intergenic | 13q14.3 | - | Cluster |
| *miR-301* | -1,12 | Intron | 17q22 | - | Single |
| *miR-25* | -1,12 | Intron | 7q22.1 | - | Cluster |
| *miR-324-5p* | -1,16 | Intron | 17q32.2 | - | Single |
| *miR-142-3p* | -1,18 | Intergenic | 17q22 | - | Single |
| *Let-7i* | -1,18 | Intergenic | 12q14.1 | + | Single |
| *miR-194* | -1,20 | Intron | 1q41 | - | Cluster |
| *miR-215* | -1,20 | Intron | 1q41 | - | Cluster |
| *miR-17-3p* | -1,22 | Intergenic | 13q31.3 | + | Cluster |
| *Let-7b* | -1,25 | Intergenic | 22q13.31 | + | Cluster |
| Continuation Table S1. | | | | | |
| ***miRNA*** | ***Relative Expression^1^*** | ***Genomic Localization*** | ***Gene Locus*** | ***DNA Strand*** | ***Organization*** |
| *miR-150* | -1,26 | Intron | 19q13.33 | - | Single |
| *miR-204* | -1,31 | Intron | 9q21.11 | - | Single |
| *miR-29c* | -1,37 | Intergenic | 1q32.2 | - | Cluster |
| *miR-302b** | -1,40 | Intron | 4q25 | - | Cluster |
| *miR-152* | -1,42 | Intron | 17q21.32 | - | Single |
| *miR-373* | -1,44 | Intergenic | 19q13.41 | + | Cluster |
| *miR-340* | -1,45 | Intron | 5q35.3 | - | Single |
| *miR-15a* | -1,45 | Intergenic | 13q14.3 | - | Cluster |
| *miR-17-5p* | -1,47 | Intergenic | 13q31.3 | + | Cluster |
| *miR-193* | -1,57 | Intergenic | 17q11.2 | + | Single |
| *miR-335* | -1,62 | Intron | 7q32.2 | + | Single |
| *miR-30e* | -1,73 | Intron | 1p34.2 | + | Cluster |
| *miR-325* | -2,12 | Intergenic | Xq21.1 | - | Single |
| *miR-26a* | -2,19 | Intron | 3p22.2 | + | Single |
| *miR-142-5p* | -2,76 | Intergenic | 17q22 | - | Single |
| *miR-107* | -3,72 | Intron | 10q23.31 | - | Single |
| ***miR-124b*** | **-5,76** | **Intergenic** | **8p23.1** | **-** | **Single** |
| ***miR-9*** | **-6,66** | **Intron** | **1q22** | **-** | **Single** |
| ***miR-200c*** | **-7,97** | **Intergenic** | **12p13.31** | **+** | **Cluster** |

(**^1^**) The numbers reported are fold values relative to the value obtained for the pool of T-ALL/CD56^-^ samples.

| Supplementary Table 2. Descriptive statistics of miR-221. | | | | |
| --- | --- | --- | --- | --- |
|  | T-cells  (n = 5) | Thymocytes  (n = 5) | T-ALL/CD56^-^  (n = 36) | T-ALL/CD56^+^  (n =12) |
| Minimum | 0.25 | 0.61 | 1.33 | 9.55 |
| 25% percentile | 0.28 | 0.70 | 3.05 | 13.87 |
| Median | 0.49 | 0.83 | 4.48 | 20.03 |
| 75% percentile | 1.05 | 1.25 | 10.05 | 32.09 |
| Mean | 0.63 | 0.95 | 7.15 | 26.44 |
| Std. Deviation | 0.41 | 0.36 | 5.23 | 17.60 |
| Std. Error | 0.18 | 0.16 | 0.87 | 5.07 |
| Lower 95% CI of mean | 0.11 | 0.49 | 5.38 | 15.26 |
| Upper 95% CI of mean | 1.14 | 1.40 | 8.92 | 37.62 |
